# Lethal Necrotizing Pneumonia Caused by an ST398 *Staphylococcus aureus* Strain

**DOI:** 10.3201/eid1706.101394

**Published:** 2011-06

**Authors:** Peter R. Davies, Elizabeth A. Wagstrom, Jeffrey B. Bender

**Affiliations:** Author affiliation: University of Minnesota, St. Paul, Minnesota, USA

**Keywords:** bacteria, pneumonia, Staphylococcus aureus, ST398, methicillin resistance, MRSA, livestock, letter

**To the Editor:** The prevalent colonization of livestock with methicillin-resistant *Staphylococcus aureus* (MRSA) sequence type (ST) 398 in many countries is a cause for consternation. However, understanding of the emergence of these organisms and their public health implications is embryonic. The perceptions that all MRSA found in livestock are of ST398 lineage or that livestock are the only reservoirs of ST398 oversimplify a complex epidemiology, therefore, prudence is required when attributing human infections with *S. aureus* ST398 to livestock reservoirs. The fatal infection of a young girl with ST398 methicillin-susceptible *S. aureus* (MSSA) is tragic (*1*). However, the conclusion by the authors that “the spread of *S. aureus* ST398 among livestock is a matter of increasing concern because strains of this sequence type were able to acquire PVL [Panton-Valentine leukocidin] genes” is misleading.

The authors report no history of livestock exposure and the *spa* type reported (t571) is relatively rare among livestock isolates (*2**,**3*). The isolate from the fatal case was tetracycline-susceptible and positive for PVL toxin, while livestock ST398 isolates have been almost uniformly tetracycline resistant and PVL negative. Notably, *spa* type t571 ST398 MSSA was detected in 9 families from the Dominican Republic living in Manhattan, New York, without contact with livestock (*4*). Furthermore, t571 was the only *spa* type of MSSA identified in a study in the Netherlands of ST398 isolates, including 3 independent cases of nosocomial bacteremia in Rotterdam with no apparent livestock contact (*5*). *spa* type t571 was the predominant (11%) MSSA type in patients at a Beijing, China, hospital (*6*). More recently, a study of t571 MSSA strains from cases of bloodstream infections in France determined that the isolates differed from pig-borne strains and shared similarities with strains from humans in China and virulent USA300 strains (*7*). These observations concur with a hypothesis that ST398 strains of diverse genotype and geographic origin may also be epidemiologically distinct (*8*), and livestock contact is a notably inconsistent feature of invasive ST398 infections (*5**,**7**–**10*).

The possibility that variants of the ST398 lineage may persist in human populations without livestock contact should not be dismissed. The incidence and severity of clinical infections with ST398 *S. aureus* in livestock workers as yet have been minimal. Understanding the public health implications of ST398 *S. aureus* requires systematic investigation of their epidemiology in animals and humans. Human clinical cases of ST398 *S. aureus* infection should not be indiscriminately attributed to livestock, particularly if isolates are genotypically dissimilar to those occurring commonly in animals.
