# Supplementary material for: Lethal Necrotizing Pneumonia Caused by an ST398 Staphylococcus aureus Strain
Source: Emerg Infect Dis. 2011 Jun;17(6):1152–3. doi: 10.3201/eid1706.101394 (PMC3358096; doi:10.3201/eid1706.101394)
Supplement: Related texts

# Reply: Lethal Necrotizing Pneumonia Caused by an ST398 *Staphylococcus aureus* Strain

**Authors:** Jean-Philippe Rasigade, Frederic Laurent, Phillipe Hubert, François Vandenesch, Jerome Etienne

**Affiliations:** Author affiliations: Hospices Civils de Lyon, Lyon, France (J.P. Rasigade, F. Laurent, J. Etienne, F. Vandenesch);; Assistance Publique–Hôpitaux de Paris, Paris, France (P. Hubert)

**In Response:** We thank Davies et al. (*1*) for their interest in our report of a lethal case of necrotizing pneumonia caused by a sequence type (ST) 398 *Staphylococcus aureus* strain (*2*). We fully agree with their request that ST398 *S. aureus* infections not be systematically attributed to contact with livestock. They correctly pointed out that several characteristics of the incriminated strain, including methicillin and tetracycline susceptibility, *spa* type, and the presence of genes encoding the Panton-Valentine leukocidin (PVL), differed from the usual genetic features of strains isolated from livestock (*3**,**4*). However, we did not state or suggest in our report that the case originated from livestock contact. Our aim in reporting this case was to warn that *S. aureus* of the ST398 lineage, regardless of its host specificity, is able to acquire PVL genes and provoke severe PVL-related infection in humans. This observation adds support to the need for controlling the increasing animal reservoir of ST398 methicillin-resistant *S. aureus* (MRSA). Indeed, the recent whole-genome analysis of an ST398 strain by Schijffelen et al. (*5*) highlighted several specific features of the ST398 genetic background, including the absence of a type I restriction and modification system. Such features have been proposed to promote horizontal gene transfer and the uptake of mobile genetic elements such as the phage-encoded PVL genes (*5*). Although phage-mediated dissemination of PVL genes into MRSA lineages does not seem to be the preeminent pathway leading to the emergence of highly epidemic PVL-positive MRSA (*6*), this eventuality should not be dismissed with respect to the ST398 lineage, which possesses all the required features to become the next MRSA “superbug” (*7*).

## References

[1] Davies PR, Wagstrom EA, Bender JB. Lethal necrotizing pneumonia caused by an ST398 *Staphylococcus aureus* strain [letter]. Emerg Infect Dis. 2011;17:1152–3. 10.3201/eid1706.10139421749803PMC3358096

[2] Rasigade JP, Laurent F, Hubert P, Vandenesch F, Etienne J. Lethal necrotizing pneumonia caused by an ST398 *Staphylococcus aureus* strain. Emerg Infect Dis. 2010;16:1330. 10.3201/eid1608.10031720678343PMC3298302

[3] Wulf M, Voss A. MRSA in livestock animals—an epidemic waiting to happen? Clin Microbiol Infect. 2008;14:519–21. 10.1111/j.1469-0691.2008.01970.x18325034

[4] van Cleef BA, Verkade EJ, Wulf MW, Buiting AG, Huijsdens XW, van Pelt W, Prevalence of livestock-associated MRSA in communities with high pig-densities in The Netherlands. PLoS ONE. 2010;5:e9385. 10.1371/journal.pone.000938520195538PMC2828479

[5] Schijffelen MJ, Boel CH, van Strijp JA, Fluit AC. Whole genome analysis of a livestock-associated methicillin-resistant *Staphylococcus aureus* ST398 isolate from a case of human endocarditis. BMC Genomics. 2010;11:376. 10.1186/1471-2164-11-37620546576PMC2900268

[6] Rasigade JP, Laurent F, Lina G, Meugnier H, Bes M, Vandenesch F, Global distribution and evolution of Panton-Valentine leukocidin-positive methicillin-susceptible *Staphylococcus aureus*, 1981–2007. J Infect Dis. 2010;201:1589–97. 10.1086/65200820367458

[7] Ferber D. Infectious disease. From pigs to people: the emergence of a new superbug. Science. 2010;329:1010–1. 10.1126/science.329.5995.101020798295
